# Exotic predators are not better biocontrol agents: the harlequin ladybird is not the most voracious in Mexico

**DOI:** 10.7717/peerj.12503

**Published:** 2021-11-16

**Authors:** Morelia Camacho-Cervantes, Wendy Mendoza-Arroyo, Daniela Arellano-Sánchez, Ek del-Val

**Affiliations:** 1 Instituto de Ciencias del Mar y Limnología, Universidad Nacional Autónoma de México, Ciudad de México, Ciudad de México, Mexico; 2 Instituto de Investigaciones en Ecosistemas y Sustentabilidad, Universidad Nacional Autónoma de México, Morelia, Michoacán, Mexico; 3 Escuela Nacional de Estudios Superiores Unidad Morelia, Universidad Nacional Autónoma de México, Morelia, Michoacán, Mexico

**Keywords:** Biocontrol, Invasive species, Pest control, Aphid, *Cycloneda sanguinea*, *Hippodamia convergens*, *Paranaemia vittigera*, Agriculture, Competition, *Harmonia axydiris*

## Abstract

The use of exotic species for pest biocontrol has been a common pathway for introduction and dispersal of invasive species that may have undesired outcomes. Biocontrol agents are believed to be a less damaging alternative than pesticides, but some species may also prey on or parasitize native species or outcompete them for resources. The harlequin ladybird (*Harmonia axyridis*) is a well-known biocontrol agent originally from Asia that has established invasive populations in 59 countries around the globe. Harlequin ladybirds are generalist predators that in addition to pests prey on an array of different species including other coccinelids’ eggs and larvae. In Mexico, native ladybirds that share ecological requirements with harlequin ladybirds are at risk of being outcompeted and predated upon. The aim of our study was to compare the foraging efficiency of harlequin ladybirds against three species of native coccinelids when preying on aphids. We investigated the foraging behaviour of ladybirds alone and in pairs with a conspecific, a native heterospecific or an exotic heterospecific. We found that the native *Cycloneda sanguinea* was the species that consumed the most aphids, while *Hippodamia convergens* was the fastest to find and consume each aphid. Harlequin ladybirds and *H. convergens* consumed the same number of aphids while *P. vittigera* consumed less. Conspecific competition was stronger than heterospecific competition. We discuss the suitability of using the exotic invasive harlequin ladybird for aphid biocontrol in comparison with native coccinelids.

## Introduction

Classical biological control is a strategy to manage pests that involves introducing natural enemies of the target pest to suppress its population (Greathead & Waage, 1983). Natural enemies are considered a great biocontrol tool to diminish arthropod pest populations, but while generalist predators are known to reduce pest numbers significantly, there is still an increasing need to understand how these exotic predators influence the communities where they are introduced (Symondson, Sunderland & Greenstone, 2002; Kenis et al., 2009). Recent studies throughout the world have found negative effects upon native Coccinellid communities (Mirande et al., 2015; Grez et al., 2016; Brown & Roy, 2018; de Castro-Guedes, de Almeida & Moura, 2020). For example, in Europe, harlequin ladybirds are recognised as at least partially responsible for the population decline of the two-spotted ladybird (*Adalia bipunctata*), following the transfer of microsporidia carried by harlequin ladybirds (Vogel, Schmidtberg & Vilcinskas, 2017).

The use of biocontrol agents can result in a reduced need for insecticide use and thus contribute to more sustainable agricultural practices (van Lenteren et al., 2018; Ali et al., 2018). In cases when a mixed approach is used, insecticides may have negative consequences for both native and exotic predator insects (Benelli et al., 2015; Dai et al., 2020; Rizwan et al., 2021). Using exotic predators as biocontrol agents could result in intraguild predation and/or competition, which may cancel out the positive effects or even result in negative effects on the diversity of native natural enemies present in the area (De Clercq, Mason & Babendreier, 2011; Mirande et al., 2015; de Castro-Guedes, de Almeida & Moura, 2020). Invasive species are considered the fourth main cause of biodiversity loss (IPBES, 2019). In North America, the introduction of exotic invasive species has long been recognized as a contributor to the decline of native species’ populations (Wheeler & Hoebeke, 1995). The many negative outcomes of invasive populations have a high toll for the global economy. In the particular case of invasive insects, a study carried out by Bradshaw et al. (2016) estimated that the negative outcomes of these insects in agriculture, health and other ecosystem services cost us at least 70 billion dollars per year.

There are many mechanisms through which exotic species displace native species. Two of the most studied are predation and competition (Simberloff et al., 2013; Blackburn, Bellard & Ricciardi, 2019). When an exotic invader establishes in an area, competition for resources may result in a decline of native species in habitats where they were formerly abundant if they are less able to exploit resources than introduced species (Smith & Gardiner, 2013). The introduction of exotic species can also have indirect effects on native communities, for example by carrying microorganisms that are lethal for native species while causing little harm to the exotic carrier (Vilcinskas et al., 2013; Amsellem et al., 2017). A successful invader is believed to possess an array of traits that collectively favour establishment and spread in novel territories (Matzek, 2012). Understanding the role exotic invaders play when competing for resources with native species is crucial to model management and conservation tools (Crookes et al., 2019). In the case of coccinellids, exotic invaders can compete with native species indirectly by consuming resources native species do, often being more efficient, or directly through predation of their larvae and eggs (Moser & Obrycki, 2009).

The harlequin ladybird *Harmonia axyridis* (Pallas) (Coleoptera: Coccinellidae) is a voracious ladybird species native to Asia that primarily feeds on aphids and is present in at least 59 countries around the globe. In some countries it was introduced as a promising biological control agent against aphids, while in other places it arrived accidentally (Koch, 2003; Lombaert et al., 2010; Roy et al., 2016; Camacho-Cervantes, Ortega-Iturriaga & del-Val, 2017). It is known for outcompeting most ladybirds in North America and Europe, and they are more resistant to parasites (Snyder, Clevenger & Eigenbrode, 2004; Koch & Galvan, 2008; Vilcinskas et al., 2013; Masetti et al., 2018). Harlequin ladybirds have been found to be more efficient when sharing the same prey with other species, such as *Hippodamia convergens* (Crookes et al., 2019), and in general have been considered a good biocontrol agent for aphids, which are among the most important crop pests globally (Dedryver, Le Ralec & Fabre, 2010; Honek et al., 2017; Masetti et al., 2018).

In Mexico, the Harlequin ladybird has been established at least since 2001. It was originally introduced in the Northern state of Chihuahua but is now established in eight or more states distributed throughout the county (Quiñones, Sánchez & Rivero, 2001; Koch, Venette & Hutchison, 2006; Goryacheva & Blekhman, 2017). We carried out this study in the State of Michoacán, where 21 species of native coccinellids have been reported (Lopez-Pina & Ponce-Saavedra, 2017). Of these species, many are abundant in agricultural fields, such as *Hippodamia convergens, Cycloneda sanguinea* and *Paranaemia vittigera* (Peña-Martínez, Coapio & Juárez, 2012; Mendoza-Arroyo, 2021). The harlequin ladybird also thrives in these agricultural fields and has been recorded predating the same aphids as the native species (M. Camacho-Cervantes, 2016, personal observations).

With this background in mind, the aim of this study was to compare the foraging efficiency of harlequin ladybirds against three species of native ladybirds (*Cycloneda sanguinea*, *Hippodamia convergens*, and *Paranaemia vittigera*) when preying on aphids alone and when paired with a conspecific, a native heterospecific or an exotic invasive heterospecific. We hypothesised that the harlequin ladybird would be more voracious and take less time to predate aphids than native species.

## Materials and Methods

### Experimental design

Adults of three native ladybirds (*H. convergens, C. sanguinea, P. vittig*era) and the exotic invasive harlequin ladybird were collected daily from cornfields in the outskirts of Morelia, Mexico (19°46′06″N, 101°11′22″O) between July 2017 and September 2017. Ladybirds were identified using a field guide (Eaton & Kaufmann, 2007). The collected ladybirds were paired by size, which had the following ranges for each species: *H. axydiris* (6.5–7 mm), *H. convergens* (6–6.5 mm), *C. sanguinea* (5.5–6 mm), *P. vittigera* (5–5.5 mm).

After being captured, individuals were kept in individual insectaries where they were not feed for a day and then observed to assess their aphid foraging efficiency (searching + handling time). Female wingless aphids (*Aphis fabae*) were also collected daily from common bean plants, which were planted in the same cornfields were ladybirds were collected. Aphid size ranged between 2 and 3 mm in length.

**Figure 1:**
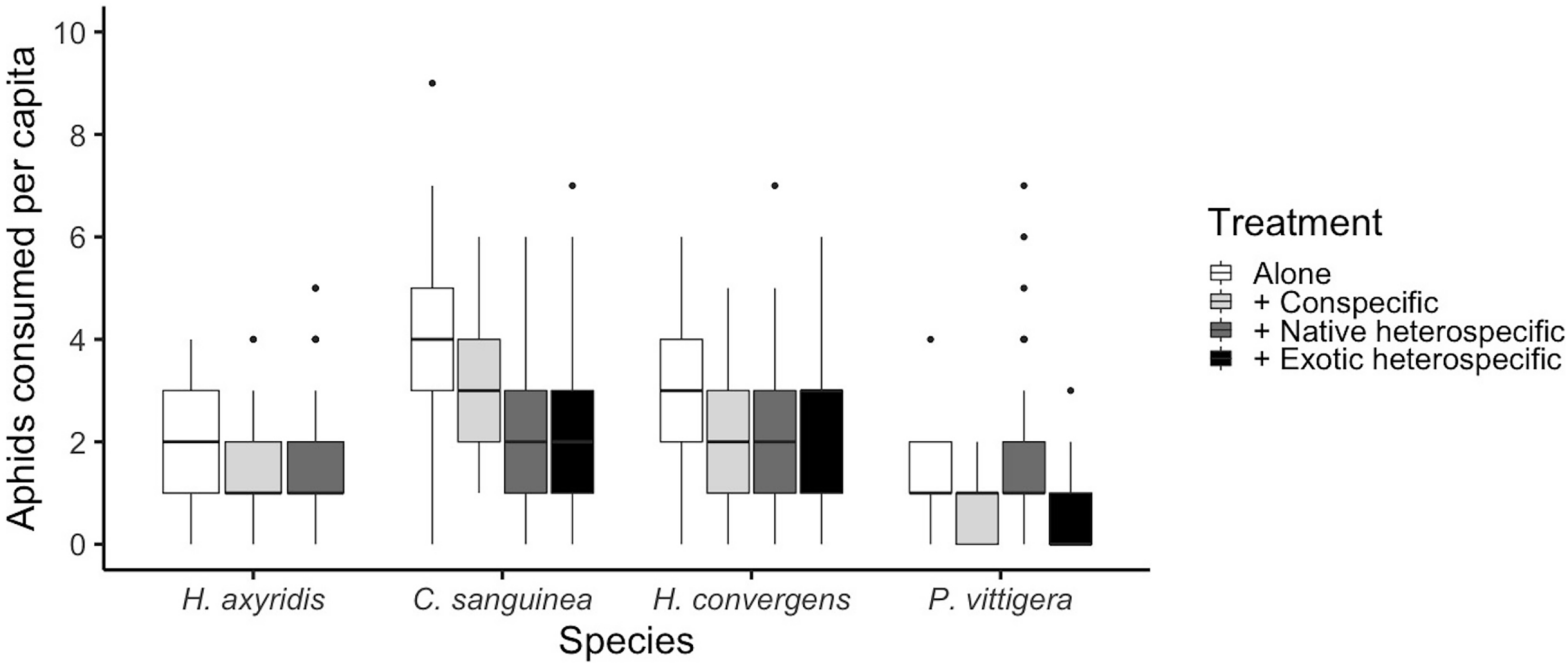
Total number of aphids consumed per species under different competition treatments. Horizontal lines represent the median, boxes indicate interquartile ranges and vertical lines show the range excluding outliers (points).

We conducted the experiments in small 250 ml transparent plastic containers that were perforated to allow transpiration. We placed a ~20 cm^2^ bean leaf and 10 aphids in the container for each observation trial. Trials were divided into four sets of treatments (1) single adult ladybird (*H. convergens, C. sanguinea, P. vittigera* or *H. axyridis*), (2) two conspecific ladybirds, (3) two heterospecific native species, and (4) a native species with the exotic invasive *H. axyridis* (see Fig. 1). This resulted in a total of 14 specific treatments: each species alone (4) + conspecific pairs (4) + native/native pairs (3; Cs/Hc, Cs/Pv, Hc/Pv) + native/exotic pairs (3; Ha/Cs + Ha/Hc + Ha/Pv). We performed 25 trials for each treatment, and each trial lasted 45 min. In paired treatments, the two individuals were recorded and analysed separately. We recorded the total number of aphids consumed per ladybird, time until the first aphid was attacked, time to consume each aphid preyed on (handling time), mean aphids consumed per replicate, and finally, search time to find the next aphid. Observations were independent, each was conducted by one observer. Observations were conducted in the morning between 9 a.m. and 12 p.m., and temperatures ranged between 19 °C and 25 °C.

### Statistical analysis

To compare the differences among treatments we performed a generalised linear mixed effects (glmer) model specifying a Poisson distribution. Our first response variable was the number of aphids consumed (a count), and the explanatory variables were competition with four levels (alone, conspecific, native heterospecific or exotic heterospecific), species with four levels (Harmonia, Cycloneda, Hippodamia, Paranaemia), and the interaction between competition and species. To test for differences in the time it took each ladybird to attack its first prey and the total time invested in each prey (search time plus handling time) we also performed glmer models with a Gamma distribution (after obtaining a *p* > 0.05 in the Kolmogorov–Smirnov test for Gamma distributions for both variables) using the same explanatory variables.

To identify differences between specific treatments we used Tukey *post hoc* tests. Each trial was assigned a unique identification number which was included as a random factor in all models to avoid pseudoreplication in cases where observations included two ladybirds, since we included the behaviour of each of the two ladybirds in our analysis. We first ran all models including interactions and when the interaction showed non-significant p value it was removed from the analysis for clarity. All of the analyses were performed using R software (R Core Team, 2020).

## Results

### Total number of aphids consumed

The number of aphids consumed was different among species (glmer: Z_3,585_ > 2.759, *p* < 0.005; Fig. 1 and Table 1). Overall, *C. sanguinea* consumed more aphids than *H. axyridis* or *P. vittigera* (Tukey: Z > 3.926, *p* < 0.001) and the same as *H. convergens* (Tukey: Z = 1.123, *p* = 142). *H. axyirids* consumed more aphids than *P. vittigera* (Tukey: Z = 2.759, *p* = 0.029), and the same as *H. convergens* (Tukey: Z = 1.892, *p* = 0.226). *P. vittigera* consumed fewer aphids than any other species (Tukey: Z > 2.759, *p* < 0.029). The number of aphids consumed by a ladybird differed among the competition treatments (alone, paired with a conspecific, with a native heterospecific, or with an exotic heterospecific; glmer: Z_3,585_ > 2.192, *p* < 0.028; Fig. 1). Ladybirds consumed more aphids when alone than when paired with a conspecific (Tukey: Z = 2.622, *p* = 0.038). There was an interaction between the competition treatment and the species (glmer: Z_8,585_ = 2.957, *p* = 0.003, Fig. 1). *P. vittigera* was the only species that consumed more aphids when with a native heterospecific than with *H. axyridis*.

**Table 1:** Mean values of aphids consumed. Number of aphids consumed, total handling time and search time per species per treatment. Mean and standard errors are shown.

Treatment	Number of aphids consumed	Time to attack the first aphid	Average handling time	Average search time
**Species**				
*H. axydiris*	1.6 ± 1.3	365.9 ± 414.9	619.5 ± 545.1	437.5 ± 379.0
*H. convergens*	2.3 ± 1.4	419.3 ± 498.1	419.7 ± 411.8	499.1 ± 431.0
*C. sanguinea*	2.9 ± 1.8	339.8 ± 439.4	436.6 ± 391.3	389.1 ± 361.5
*P. vittigera*	1.1 ± 1.2	443.4 ± 539.5	637.8 ± 520.0	521.9 ± 512.7
**Competition**				
Alone	3.4 ± 1.8	309.8 ± 345.0	341.9 ± 420.7	430.1 ± 277.2
Conspecific	2.1 ± 1.4	485.1 ± 547.9	441.5 ± 525.9	521.4 ± 489.8
Native heterospecific	2.3 ± 1.5	347.7 ± 469.8	540.2 ± 584.9	432.2 ± 441.3
Exotic heterospecific	2.8 ± 1.7	369.3 ± 368.1	449.8 ± 475.8	402.1 ± 291.3

### Time to attack the first aphid

**Figure 2:**
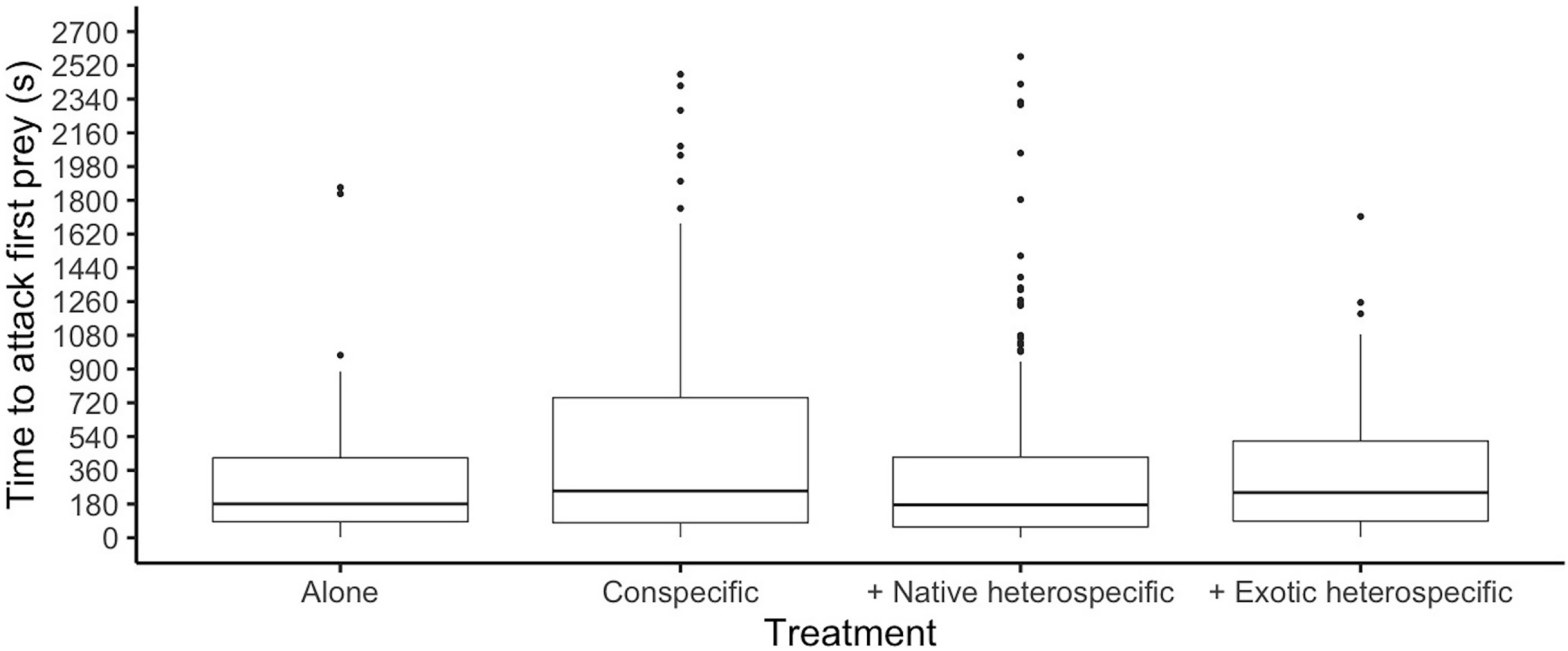
Time it took ladybirds to attack its first prey in different treatments. Horizontal lines represent the median, boxes indicate interquartile ranges and vertical lines show the range excluding outliers (points).

The time it took each ladybird to attack its first prey was similar among species (glmer: Z_8,497_ < 1.161, *p* > 0.245) and was significantly different among competition treatments (glmer: F_3,497_ = 3.91, *p* = 0.006, Fig. 2). Ladybirds alone attacked their first prey faster than when interacting with a conspecific (Tukey: Z = 3.205, *p* = 0.007) and attacked faster when paired with a native heterospecific than with a conspecific (Tukey: Z > 2.827, *p* < 0.023). The interaction between species and competition treatments was not significant (*p* > 0.05).

### Total time per aphid consumed

**Figure 3:**
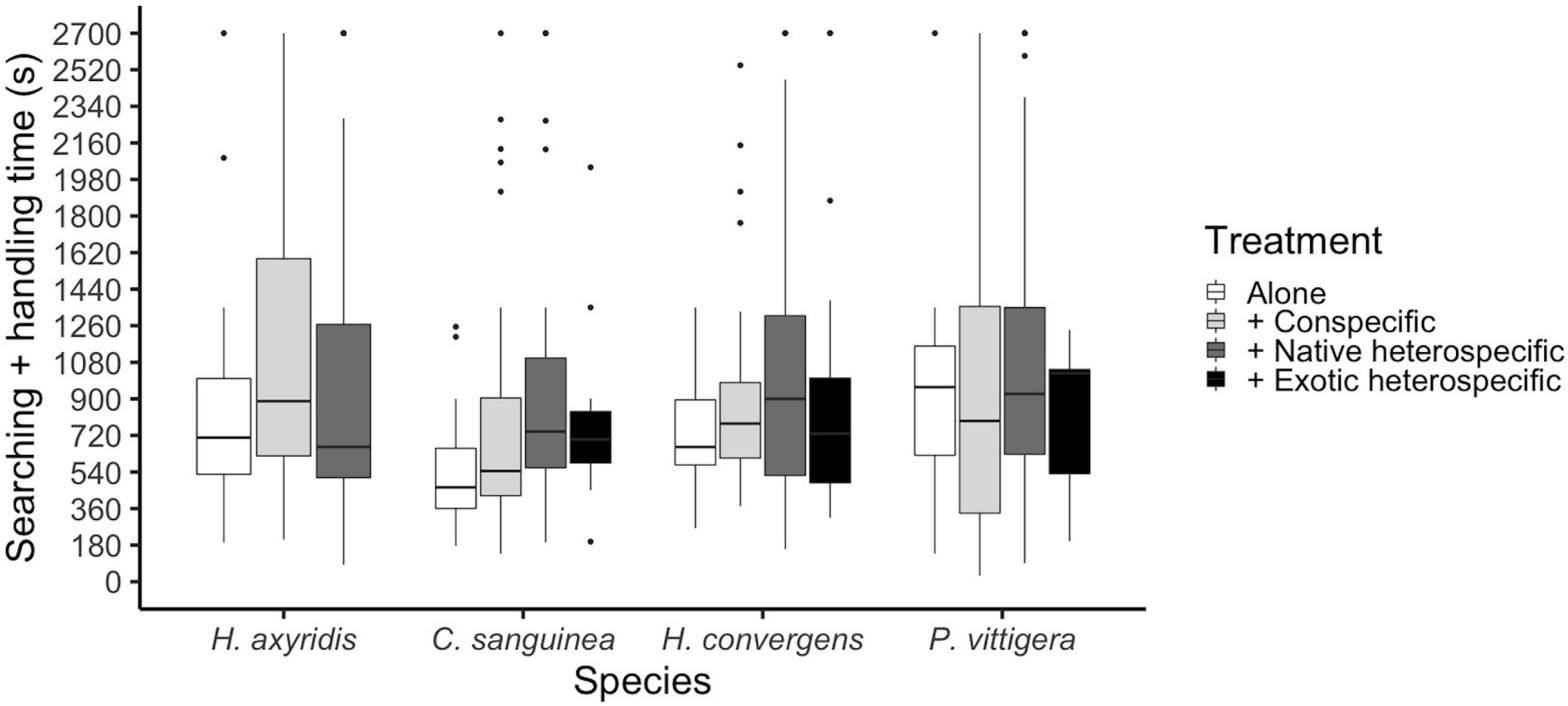
Ladybird average invested time per aphid, calculated as the sum of the time it took to find an aphid plus the time it took it to finish it and start a new search. Horizontal lines represent the median, boxes indicate interquartile ranges and vertical lines show the range excluding outliers (points).

The total time a ladybird took to consume an aphid was calculated as the sum of the searching time plus the time it took it to consume the aphid and start a new search. This time period differed among species (glmer: Z_3,475_ = 2.055, *p* = 0.039); *P. vittigera* took longer than *C. sanguinea* (Tukey: *Z* = 2.614, *p* < 0.044). This time was similar among competition treatments overall (glmer: Z_3,404_ < 1.399, *p* = 0.162, Fig. 3), but there was a significant interaction between species and competition treatment (glmer: Z_3,475_ = 2.413, *p* = 0.016) in which *C. sanguinea* took less time per aphid when alone than when with a conspecific, when the rest of the species took the same amount of time in all treatments.

## Discussion

The harlequin ladybird has a long history of use as a classical biological control agent against aphids (Camacho-Cervantes, Ortega-Iturriaga & del-Val, 2017). We found that, at least in west-central Mexico, this species might not be the best option to control aphids; it is neither the most voracious nor the most efficient predator compared to the native species we tested. Although it was no stronger a competitor for native ladybirds than native heterospecifics, harlequin ladybirds represent a threat to native coccinellids in multiple other ways. We will further discuss our results by focusing first on their competition effects, and then on their use as biocontrol for aphids.

### Competition effects

Harlequin ladybirds are generalist voracious predators widely used as biocontrol agents that are considered a threat to local species. Since it was first introduced outside its native range, it is now present in 59 countries worldwide (Brown et al., 2011; Camacho-Cervantes, Ortega-Iturriaga & del-Val, 2017). In Mexico it was first illegally introduced around 2001, since that date it is now present in 23 of 31 Mexican states (Enciclovida), however its impacts on local biodiversity are unknown (Quiñones, Sánchez & Rivero, 2001; Koch, 2003; Goryacheva & Blekhman, 2017).

One of the main mechanisms associated with the increasing success of exotic invasive species in the new region is competition with native species (Blackburn, Bellard & Ricciardi, 2019). Therefore, our study investigated whether the exotic harlequin ladybird was a superior competitor to native species. When comparing the number of aphids consumed per ladybird, the native *C. sanguinea* was the most voracious, while the harlequin ladybird and *H. convergens* were intermediate and *P. vittigera* consumed the fewest aphids. In other words, the exotic species was only a better competitor in terms of food intake against one of the native species. This pattern was also observed in the UK when comparing the feeding rate of harlequin ladybird larvae competing against larvae of the native species *A. bipunctata* and *C. septempunctata* (Hentley et al., 2016). *A. bipunctata* increased food intake when competing with harlequin ladybirds, while *C. septempunctata* fed as if it was alone. Thus, it appears that some native species are faster to consume aphids under the experimental conditions evaluated.

Regarding the total handling time (search time plus consumption time), the ranking of the species was similar to in total aphid consumption; *C. sanguinea* was the fastest followed by the harlequin ladybird and *H. convergens*, while *P. vittiger*a took longer to search for and consume aphids. A recent study by Crookes et al. (2019) also found that the exotic invasive harlequin ladybird is faster than the native *H. convergens*. This could suggest a significant advantage of harlequin ladybirds when preying in the same fields against some native species such as *H. convergens and P. vittigera*, but not against *C. sanguinea*.

When foraging in pairs, we found the strongest competition effects when ladybirds were paired with conspecifics. All individuals took longer to attack their first prey and consumed fewer aphids than when alone when paired with a conspecific ladybird. When paired with a heterospecific, *P. vittigera* was the only species that showed lower total consumption of aphids when accompanied by the exotic invasive harlequin ladybird, while the other species consumed the same number of aphids when accompanied by a native heterospecific or by the harlequin ladybird. In contrast to our results, Zaviezo, Soares & Grez (2019) found that harlequin ladybirds were more voracious when sharing resources with the exotic *H. variegata* and native *E. chilensis*. More like our results, Bertleff et al. (2021) found in Germany that the harlequin ladybird is as voracious as the native *C. septempunctata* but more voracious than other smaller native ladybirds. They argue that this difference is due to size of the ladybirds. In our study, harlequin ladybirds behaved very similar to *H. convergens* but did not show higher efficiency in any of the recorded traits. Still, our study was designed to test for the foraging efficiency of adult ladybirds. Even though the harlequin ladybird’s distributional range is expanding in Mexico, it appears that several Mexican Coccinellids might be more voracious than them. We acknowledge that the patterns we report may not hold under field conditions, as has been found when comparing larval behaviours (Roy et al., 2016) and volatile attraction (Rondoni et al., 2017) among others; these aspects warrant further investigation in our study region.

### Biocontrol

Biocontrol agents for pest control are thought to be a more sustainable alternative than pesticides in agriculture. However, when exotic species are used, several investigations have shown negative results (Symondson, Sunderland & Greenstone, 2002; Louda et al., 2003; De Clercq, Mason & Babendreier, 2011; Van Driesche & Hoddle, 2016). The harlequin ladybird has been introduced outside its native range with the intention of avoiding or reducing pesticide use, but the negative effects of its introduction have outweighed the benefits in terms of pest control in several places (Snyder, Clevenger & Eigenbrode, 2004; Vilcinskas et al., 2013; Masetti et al., 2018).

Farmers in Mexico are keen to control aphids in their crops using natural enemies as biocontrol in an attempt to achieve more sustainable practices, and the harlequin ladybird is not yet considered noxious (E. del-Val, 2016, personal observations). The use of an augmentative biological control or conservation biological control strategy using local native coccinellid species instead of the exotic harlequin ladybird may be a better strategy to control aphids in crops. Although detrimental effects of *H. axydiris* upon biodiversity in Mexico are not yet evident, this hypothesis needs to be further explored in the field and results must be shared with local stakeholders to enhance invasion awareness. Our results provide some evidence that local coccinellid communities (at least in central Mexico) could have species that are better aphid predators than harlequin ladybirds. Since the harlequin ladybird is not the most voracious species compared to other native ladybirds in the region, native species like *C. sanguinea* may be a better biocontrol option, both in terms of being more efficient a voracious and in terms of avoiding the threat that exotic species can pose to biodiversity. To increase native coccinellid abundances, we suggest a conservation biological control approach by improving crop field margins (Begg et al., 2017; Landis, Wratten & Gurr, 2000). In this particular case, native species might deliver the same (or even stronger) benefits than exotic predator species when used to control pests, and native coccinellid species are still abundant in Mexico.

## Conclusions

The harlequin ladybird is not the most efficient predator of aphids when compared with three native species from west-central Mexico. Even if widely used in the area, this species might not be the best option for aphid control, *Cycloneda sanguinea* and *Hippodamia convergens* proved to be equally or more efficient than the harlequin ladybird (*i.e*. they eat the same amount of aphids at the same or faster rate), and their use would avoid the negative effects associated with the introduction or reproduction of exotic species as biocontrol. When competing with native coccinellids, harlequin ladybirds posed similar competition to a native heterospecific, but the introduction of this species has proved to be detrimental for native species in multiple other ways in other studies. We therefore suggest shifting focus to conservation biological control (Landis, Wratten & Gurr, 2000), by improving vegetation in agricultural field margins so that native Coccinellid populations will be able to thrive in intensive agriculture fields and increase their biocontrol capacities (Begg et al., 2017). Biocontrol should be as prey-specific as possible, and it is preferred the use of native species after a careful local assessment before releasing individuals to avoid negative side effects.

## Supplemental Information

10.7717/peerj.12503/supp-1 Supplemental Information 1 Time invested on different activities by ladybirds on the different experiments. The different replicates specifying the treatments with four explanatory variables: 1) time to attack the first prey (sec), 2) minimal time to attach the first prey (sec), 3) average handling time (sec), 4) average searching time (sec), 5) total time per aphid (sec), 6) total number of aphids consumed (number of individuals). All variables were used to analyse differences between competition, between species and the interaction between competition and species.

## Additional Information and Declarations

### Data Availability

The following information was supplied regarding data availability: The raw data is available in the Supplemental File.
